# High Prevalence of Antibiotic Resistance in Iranian *Helicobacter pylori* Isolates: Importance of Functional and Mutational Analysis of Resistance Genes and Virulence Genotyping

**DOI:** 10.3390/jcm8112004

**Published:** 2019-11-17

**Authors:** Nastaran Farzi, Abbas Yadegar, Amir Sadeghi, Hamid Asadzadeh Aghdaei, Sinéad Marian Smith, Josette Raymond, Hidekazu Suzuki, Mohammad Reza Zali

**Affiliations:** 1 Foodborne and Waterborne Diseases Research Center, Research Institute for Gastroenterology and Liver Diseases, Shahid Beheshti University of Medical Sciences, Tehran 1985717411, Iran; nastaran_farzi@yahoo.com; 2 Gastroenterology and Liver Diseases Research Center, Research Institute for Gastroenterology and Liver Diseases, Shahid Beheshti University of Medical Sciences, Tehran 1985717411, Iran; amirsadeghimd@yahoo.com (A.S.); nnzali@hotmail.com (M.R.Z.); 3 Basic and Molecular Epidemiology of Gastrointestinal Disorders Research Center, Research Institute for Gastroenterology and Liver Diseases, Shahid Beheshti University of Medical Sciences, Tehran 1985717411, Iran; hamid.assadzadeh@gmail.com; 4 School of Medicine & School of Pharmacy and Pharmaceutical Sciences, Trinity College Dublin, Dublin 2, Ireland; smithsi@tcd.ie; 5 Bacteriology, University of Paris-Descartes, Cochin Hospital, 75006 Paris, France; josette.raymond@aphp.fr; 6 Division of Gastroenterology and Hepatology, Department of Internal Medicine, Tokai University School of Medicine, 143 Shimokasuya, Isehara, Kanagawa 259-1193, Japan; hsuzuki@tokai.ac.jp

**Keywords:** *Helicobacter pylori*, resistance genes, mutations, virulence genotype, *cag*PAI intactness

## Abstract

The high prevalence of antibiotic resistance in *Helicobacter pylori* has become a great challenge in Iran. The genetic mutations that contribute to the resistance have yet to be precisely identified. This study aimed to investigate the prevalence of antibiotic resistance and virulence markers in Iranian *H. pylori* isolates and to analyze if there is any association between resistance and genotype. Antibiotic susceptibility patterns of 68 *H. pylori* isolates were investigated against metronidazole, clarithromycin, amoxicillin, rifampicin, ciprofloxacin, levofloxacin, and tetracycline by the agar dilution method. The *frxA*, *rdxA*, *gyrA*, *gyrB*, and 23S rRNA genes of the isolates were sequenced. The virulence genotypes were also determined using PCR. Metronidazole resistance was present in 82.4% of the isolates, followed by clarithromycin (33.8%), ciprofloxacin (33.8%), rifampicin (32.4%), amoxicillin (30.9%), levofloxacin (27.9%), and tetracycline (4.4%). Overall, 75% of the isolates were resistant to at least two antibiotics tested and considered as a multidrug resistance (MDR) phenotype. Most of the metronidazole-resistant isolates carried frameshift mutations in both *frxA* and *rdxA* genes, and premature termination occurred in positions Q5Stop and Q50Stop, respectively. Amino acid substitutions M191I, G208E, and V199A were predominantly found in *gyrA* gene of fluoroquinolone-resistant isolates. A2143G and C2195T mutations of 23S rRNA were found in four clarithromycin-resistant isolates. Interestingly, significant associations were found between resistance to metronidazole (MNZ) and *cagA*-, *sabA*-, and *dupA*-positive genotypes, with *p* = 0.0002, *p* = 0.0001, and *p* = 0.0001, respectively. Furthermore, a significant association was found between *oipA* “on” status and resistance to amoxicillin (AMX) (*p* = 0.02). The prevalence of *H. pylori* antibiotic resistance is high in our region, particularly that of metronidazole, clarithromycin, ciprofloxacin, and MDR. Simultaneous screening of virulence and resistance genotypes can help clinicians to choose the appropriate therapeutic regime against *H. pylori* infection.

## 1. Introduction

*Helicobacter pylori* (*H. pylori*) is known as the most common human pathogen infecting more than half of the world’s population [1,2]. Early eradication-based therapies have been proven to regress the *H. pylori*-associated diseases [3,4]. However, the efficacy of eradication treatments has been extremely compromised primarily because of increased resistance to antimicrobial agents in many countries [5,6,7,8].

Today, first-line standard triple therapy is the most widely used eradication treatment for *H. pylori* infection, which typically comprises two of three antibiotics including amoxicillin, clarithromycin, and metronidazole in combination with one proton pump inhibitor (PPI) [3,9]. However, the uses of levofloxacin or ciprofloxacin in fluoroquinolone containing triple therapy and bismuth-based quadruple therapy have also been suggested as second-line therapies after the failure of the clarithromycin-containing regimens [10,11,12]. Furthermore, tetracycline and rifampicin are among the common antibiotics that have been used in several rescue therapies recommended in the eradication of *H. pylori* infection [13,14,15].

Previous studies have demonstrated that numerous point mutations resulting from genetic plasticity within the chromosomal genes are the main antibiotic resistance mechanism among *H. pylori* strains in various geographic regions [5,6,16,17,18]. Primary resistance to clarithromycin has been mainly associated with point mutations in the peptidyl transferase region encoded in domain V of 23S rRNA. Most of these mutations include nucleotide substitutions involving an adenine to guanine transition at positions 2142 and 2143 and, to a lesser extent, an adenine to cytosine transversion at position 2142 [8,10,19]. However, several other mutations associated with clarithromycin resistant isolates seem to be emerging [20,21]. The mechanisms of metronidazole resistance in *H. pylori* are frequently attributed to inactivating mutations in *rdxA* and *frxA* genes [22,23]. On the other hand, mutational changes leading to various amino acid substitutions that confer fluoroquinolone resistance have been located in different positions of the quinolone-resistant determining region (QRDR) of *gyrA* and *gyrB* genes [19,24].

Apart from the aforementioned mechanisms of resistance developed by *H. pylori* strains to the major antibiotics used in the treatment of infection, other factors such as the virulence genotype status of bacteria have been reported to affect drug resistance [25,26,27,28,29]. However, the exact underlying mechanisms involved in the crosstalk of *H. pylori* virulence and antimicrobial resistance remained to be clarified.

Hence, the focus of the present study was to evaluate the antibiotic susceptibility patterns and underlying resistance mechanisms of *H. pylori* strains isolated from Iranian patients with different gastric diseases. Furthermore, we determined the presence of genetic mutations that are associated with antibiotic resistance. We also examined the possible association between resistance profiles and a panel of virulence genotypes.

## 2. Materials and Methods

### 2.1. Patients and H. pylori Isolates

Antral biopsies were collected for culture from 160 patients who underwent upper gastroduodenal endoscopy at Taleghani Hospital in Tehran from February 2016 to August 2017. Patients were excluded if they were taking eradication therapy for *H. pylori*, PPIs, or H_2_-receptor blockers, and any antibiotics used for other infections within two weeks prior to enrolment. The study protocol was approved by the Ethical Review Committee of the Research Institute for Gastroenterology and Liver Diseases at Shahid Beheshti University of Medical Sciences (Project No. IR.SBMU.RIGLD.REC.1395.878). All experiments were performed in accordance with relevant guidelines and regulations recommended by the institution and informed consents were obtained from all subjects and/or their legal guardians prior to sample collection.

The biopsy specimens were smeared on Brucella agar (Merck, Darmstadt, Germany) plates containing 7% horse blood (v/v), 10% fetal calf serum (FCS), Campylobacter supplement (Skirrow, Quelab, Montreal, Quebec, Canada), and amphotericin B (2.5 mg/L). The inoculated plates were incubated at 37 °C in a CO_2_ incubator under microaerophilic atmosphere containing approximately 5% O_2_, 10% CO_2_, and 85% N_2_ for 3–7 days. The *H. pylori* was identified by colony and microscopic morphology, positive catalase, oxidase, and urease tests, and confirmed by molecular assays [30,31,32].

### 2.2. Antibiotic Susceptibility Testing

The antibiotic susceptibility of the *H. pylori* strains was assessed by the agar dilution method against a panel of seven antibiotics purchased from Sigma-Aldrich (St. Louis, MO, USA), including metronidazole (MNZ), clarithromycin (CLR), amoxicillin (AMX), rifampicin (RIF), ciprofloxacin (CIP), levofloxacin (LEV), and tetracycline (TCN). The range of antibiotic concentrations was as follows: 0.25–256 mg/L for MNZ, 0.06 to 64 mg/L for CLR, 0.03 to 4 mg/L for AMX, 0.03 to 32 mg/L for RIF and LEV, 0.06 to 32 mg/L for CIP, and 0.06 to 16 mg/L for TCN. *H. pylori* inoculums were prepared from 72 h old cultures that were suspended in sterile saline and adjusted to a density equal to No. 3 McFarland standard. The bacterial suspensions were inoculated directly onto Mueller–Hinton blood agar (Merck, Darmstadt, Germany) plates supplemented with 10% defibrinated horse blood containing antibiotic dilutions, and were incubated under microaerophilic conditions, as over-mentioned. After 72 hours of incubation, the minimal inhibition concentrations (MICs) were determined as the lowest concentration of antibiotic that completely inhibited the growth of the inoculums. The resistance breakpoints were used as described by the last guideline of European Committee on Antimicrobial Susceptibility Testing (EUCAST version 8.0, http://www.eucast.org/). Strains were considered to be resistant for MICs of >8 mg/L for MNZ; >0.125 mg/L for AMX; and >1 mg/L for RIF, CIP, LEV, and TCN. Clarithromycin MICs were interpreted based on CLSI breakpoints (≤0.25 mg/L, susceptible; 0.5 mg/L, intermediate; ≥1.0 mg/L, resistant) [33]. *H. pylori* strains with resistance to at least two antimicrobial agents were considered as isolates with the multidrug resistance (MDR) phenotype [34]. A clinical isolate of *H. pylori* with previously identified MIC values served as a quality control strain in all susceptibility tests [35].

### 2.3. Genomic DNA Extraction

Subcultures of the single colonies were prepared, and confluent cultures from each colony were used for DNA extraction using QIAamp DNA extraction kit (QIAgen®, Hilden, Germany), following the manufacturer’s directions. The DNA samples were stored at −20 °C until used for gene amplification.

### 2.4. Mutation Analysis of the Resistance Genes

To detect specific mutations in the *frxA*, *rdxA*, *gyrA*, *gyrB*, and 23S rRNA genes, a PCR-based sequencing approach was carried out for a selected number of *H. pylori* isolates, including the susceptible and resistant strains. The oligonucleotide primers are shown in Table 1. The PCR products were sequenced on both strands by the Sanger sequencing method using an automated sequencer (Macrogen, Seoul, Korea). All complete and partial DNA sequences were edited by Chromas Lite version 2.5.1 (Technelysium Pty Ltd, Australia). Comparative sequence analysis between resistant and sensitive strains was carried out using BioEdit software version 7.2.5 [36]. The DNA and deduced amino acid sequences were aligned and coordinated to *H. pylori* 26695 (GenBank: CP003904.1) as a reference sequence.

### 2.5. Detection of Virulence Markers

The presence of virulence factors including *cagA*, *cagL*, and *vacA* alleles (s1/s2 and m1/m2), as well as *babA2*, *sabA*, and *dupA* genes, were assessed by PCR [37,38]. The diversity of the *cagA* C-terminal variable region, the functional (on/off) status of *oipA* gene, and the intactness of the *cag*PAI locus was also analyzed by the PCR-sequencing method [30,39,40]. *H. pylori* J99 (CCUG 47164) and a no-template reaction were used as positive and negative controls in all amplifications, respectively. The sequences of oligonucleotide primers are presented in Table 1.

### 2.6. Nucleotide Sequence Accession Numbers

The sequences obtained from this study were submitted to NCBI under the following GenBank accession numbers: domain V 23S rRNA, MH040926-MH040949; *gyrA*, MH054262-MH054292; *gyrB*, MH054293-MH054319; *frxA*, MH054320-MH054346; *rdxA*, MH054347-MH054374.

### 2.7. Statistical Analysis

The SPSS Statistics for Windows (version 21.0, IBM Corp, Armonk, NY, USA) was used to perform all statistical analyses. Chi-square and Fisher’s exact tests were used to determine the statistical significance of differences between categorical variables. A *p*-value of less than 0.05 was considered as statistically significant.

## 3. Results

### 3.1. Characteristics of Patients

Totally, 68 (42.5%) *H. pylori* isolates were cultured from antral biopsies of the patients included in the study. The *H. pylori* infected patients consisted of 31 (45.6%) men and 37 (54.4%) women, with an average age of 46.5 ± 8.3 years old (range 23–75 years). Endoscopic diagnosis showed that 43 patients had chronic gastritis (CG), 18 had peptic ulcer disease (PUD), and 7 had intestinal metaplasia (IM).

### 3.2. Prevalence of Antibiotic Resistance

Overall, the metronidazole resistant detection rate was 82.4% (56/68), and the lowest resistance rate was observed against tetracycline in 3/68 (4.4%) isolates. Resistance to clarithromycin, rifampicin, and amoxicillin was observed in 23/68 (33.8%), 22/68 (32.4%), and 21/68 (30.9%) of isolates, respectively. Five (7.4%) isolates were found as intermediate to clarithromycin. *H. pylori* resistance to ciprofloxacin and levofloxacin was detected in 23/68 (33.8%) and 19/68 (27.9%) of isolates, respectively. Only one isolate was found to be susceptible to all antibiotics examined. The rate of resistance to metronidazole, clarithromycin, amoxicillin, and levofloxacin was higher in patients with PUD and IM than with CG patients. Inversely, the rate of resistance to rifampicin was higher in CG patients than with PUD and IM. There were no important differences in the rate of resistance to ciprofloxacin and tetracycline between CG and with PUD and IM patients. All patients with IM were resistant to metronidazole. The MIC range, MIC50/MIC90, prevalence of resistance, and distribution of MIC values for the *H. pylori* strains are shown in Table 2 and Table 3.

### 3.3. Rate of MDR Phenotype

Single-drug resistance (SDR) was observed in 16/68 (23.5%) isolates, in which resistance to metronidazole was the most frequent SDR phenotype (9/16, 56.2%). Totally, 51/68 (75%) isolates showed multidrug resistance (MDR) phenotype, and 17 different MDR profiles were detected. No isolate was resistant to all tested antibiotics. The distribution of the SDR and MDR profiles within various clinical diagnosis groups is shown in Table 4. Almost all of the isolates from patients with IM and most of the PUD isolates showed an MDR phenotype. Resistance to MNZ + RIF was the most common double-drug resistance profile (6/20, 30%). Resistance to MNZ was found in all isolates with triple-drug and quadruple-drug resistance profiles. Resistance to TCN was only found in isolates with quadruple-drug resistance profiles.

### 3.4. Genetic Variations of frxA and rdxA Genes

Totally, 27 of the *frxA* and 28 of the *rdxA* genes obtained from all isolates were sequenced and analyzed, as shown in Table 5. Fourteen (51.8%) isolates exhibiting resistance to metronidazole predominantly carried insertions and/or deletions resulting in translational frameshift mutations in the FrxA. One isolate was found to have a stop codon at position Q5Stop, resulting in premature termination codon (PTC), while missense mutations were found in 11/27 (40.7%) isolates. In addition, about one-third (10/28, 35.7%) of the isolates were found to have frameshift mutations in the *rdxA* gene. Nonsense mutations resulting in PTC were identified in 3/28 (10.7%) isolates owing to codon substitutions at position Q50Stop of RdxA. Missense mutations were distributed among 5 susceptible and 10 resistant isolates of the *rdxA* genes. One resistant strain had no mutation in both genes. The peptide sequence alignments for the *frxA* and *rdxA* genes from metronidazole-susceptible and -resistant isolates in comparison with the reference strain are presented in Supplementary Figures S1 and S2, respectively.

### 3.5. Amino Acid Variations at the QRDR Region of gyrA and gyrB Genes

As shown in Table 6 and Supplementary Figures S3 and S4, selective regions in the QRDR of *gyrA* and *gyrB* genes were sequenced among 31 and 27 *H. pylori* isolates, respectively. Totally, 16 different amino acid substitutions were detected in *gyrA* subunit among all isolates. Three different amino acid variants including S63P, R140K, and A183V were detected to be exclusively present in *gyrA* of the fluoroquinolone-resistant isolates, whereas six different substitutions of A97V, D143E, A207T, G208K, I212S, and E214K were found to be present in the susceptible isolates only. In addition, seven other mutations were observed at D86N, D86G, V150A, M191I, V199A, G208A, and G208E in both fluoroquinolone-susceptible and -resistant isolates. The most frequent substitutions in *gyrA* of the fluoroquinolone-resistant isolates were M191I (14/23, 60.9%), G208E (13/25, 52%), and V199A (5/9, 55.5%), respectively. The M191I-G208E substitution was found to be the most common double mutation (8/12, 66.7%) within five resistant and three susceptible isolates, while the M191I-V199A-G208E was found as the most frequent triple substitutions (3/9, 33.3%) from two susceptible and one resistant isolates. The quadruple substitution was detected in *gyrA* of three resistant and three susceptible isolates.

As for the *gyrB* subunit, two different amino acid variants including D481E and R484K were detected among seven isolates. The D481E substitution was found to be present in both fluoroquinolone-susceptible and -resistant isolates, whereas R484K was exclusively present in resistant isolates. As shown in Table 6 and Supplementary Figure S4, two fluoroquinolone-susceptible isolates had the single D481E mutations, while five resistant isolates presented the double D481E-R484K only. No mutation of *gyrB* was found in 11 fluoroquinolone-resistant and 9 susceptible isolates.

### 3.6. Genetic Variations of the 23S rRNA Gene

The domain V of the 23S rRNA gene was sequenced in 24 *H. pylori* isolates. As shown in Supplementary Figure S5, this region was highly conserved with minimal nucleotide variations in comparison with *H. pylori* strain 26695 as the reference genome. Overall, four nucleotide transitions including A2143G and C2195T were identified in clarithromycin-resistant isolates. None of these mutations were observed among the susceptible isolates and no isolates were found to have double mutations of A2143G and C2195T. The distribution of MIC values according to the different mutations in all phenotypically resistant and susceptible isolates is presented in Table 7.

### 3.7. Association between Virulence Genotypes and Resistance Patterns

The frequency and distribution of strains grouped by virulence genotypes according to each susceptibility pattern are shown in Table 8. *cagL*-positive genotype was detected in all resistant isolates to CLR, CIP, LEV, RIF, and TCN. All resistant isolates to TCN were found to have the *cagA*-positive genotype. There was a significant association between the presence of *cagA* and resistance to MNZ (*p* = 0.0002). The *cagA* ABC motif was also found frequently in susceptible isolates, with the exception of metronidazole. The *cagA* ABCCC motif was detected in all resistant isolates to MNZ, AMX, and CIP. While no significant association was found between *cag*PAI integrity and antibiotic resistance (*p* > 0.05), most of the resistant isolates and all isolates resistant to TCN carried an intact *cag*PAI locus. *H. pylori* isolates with *vacA* s1m2 were found more frequently in resistant isolates to all antibiotics tested. Strains with *oipA* “on” status and *babA2*, *sabA*, and *dupA* positivity were also frequently found in resistant isolates. A significant association was found between *oipA* “on” status and resistance to AMX (*p* = 0.02). *babA2*-positive genotype was detected in all intermediate and resistant isolates to CLR, as well as in all isolates resistant to LEV and TCN. *sabA*-positive genotype was detected in all resistant isolates to TCN, and this genotype was significantly associated with resistance to MNZ (*p* = 0.0001). Further, there was a statistically significant association between *dupA*-positivity and resistance to MNZ (*p* = 0.0001).

## 4. Discussion

Eradication of *H. pylori* infection has been decreasing progressively, mainly because of increased resistance to antimicrobial agents, especially in developing countries [5,6,44,45,46,47]. In Iran, nearly 40–90% of the adult population is infected with *H. pylori*, which seems to be acquired early in childhood [35,37,48]. There have been few reports on the antibiotic resistance of *H. pylori* in Iran by performing the agar dilution method as the reference method for this bacterium [35,49,50,51]. Therefore, we carried out this work to determine the phenotypic and molecular characteristics of *H. pylori* antibiotic resistance, and to evaluate the association between resistance patterns and a wide panel of virulence genotypes. The prevalence of metronidazole resistance was reported to be high among Iranian *H. pylori* strains and ranged from 40.5% to 78.6% [49,51,52]. The results of this study showed an increased rate of metronidazole resistance (84.4%) as compared with previous reports from Iran [35,49,51]. A very high prevalence of metronidazole resistance has also been reported from other developing countries in Asia, including Bangladesh (77.5%), China (95.4%), India (83.8%), Kuwait (70%), Pakistan (89%), and Vietnam (69.9%) [47,53,54,55,56,57]. The extremely high rate of metronidazole resistance observed in this study might be attributed to the widespread and unauthorized consumption of antimicrobial drugs in Iran. In addition, massive use of metronidazole in the treatment of various infections such as anaerobic bacterial and parasitic infections, as well as for diarrheal, dental, periodontal, and gynecologic diseases, could explain the significantly high rate of metronidazole resistance in many developing countries [5,35,45,47]. Therefore, in agreement with other previous studies, *H. pylori* treatment regimens containing metronidazole are not useful and should not be chosen as first-line eradication therapy in Iran [5,45,46,47,58].

Previous studies demonstrated that various point mutations in *frxA* and *rdxA* genes were linked to metronidazole resistance in *H. pylori* [23,55,59]. As expected, different types of mutations including insertions, deletions, missense, nonsense, and frameshift mutations were detected among the studied strains. Our results showed that most of the metronidazole-resistant isolates presented frameshift mutations in these genes. Moreover, we found point mutations introducing stop codon at positions Q5Stop and Q50Stop in the *frxA* and *rdxA* genes, respectively. Many other nonsense mutations that lead to PTC have also been reported in *rdxA* and rarely in *frxA* genes [5,17,22,41,45,60,61]. However, in this study, one of the metronidazole-resistant isolate did not contain any alterations in both the *frxA* and *rdxA* genes. As previously suggested, metronidazole resistance in this small subset of isolates may be the result of the presence of additional resistance mechanisms and mutations in other redox enzymes [41,45,62].

Fluoroquinolones were proven to have bacteriostatic activities by trapping DNA gyrase and topoisomerase IV. These drugs are considered as a salvage treatment for *H. pylori* eradication in second- or third-line therapies after the failure of clarithromycin-based treatment regimens [12,24,63]. However, it has been reported that fluoroquinolone resistance is rapidly expanding around the world [6,7,57]. In a previous study from Iran, the rate of resistance to ciprofloxacin and levofloxacin was reported to be about 27% and 24.3%, respectively [35]. In this study, we found a significant increase of fluoroquinolone resistance, which is of great concern. Nevertheless, studies from Taiwanese and Malaysian populations revealed that gemifloxacin is superior to levofloxacin in antimicrobial activity and may have better drug efficacy than levofloxacin in *H. pylori* eradication [45,64].

Point mutations in the QRDR of *gyrA* and *gyrB* sequences greatly reduce the antimicrobial activity of fluoroquinolones. To date, several mutations have been identified in the *gyrA* subunit of *H. pylori* strains from different geographical regions [5,8,42,45,64,65,66,67,68]. None of the most common mutations in *gyrA* hot spot positions N87K, D91N, and D91G were detected in our isolates. However, 10 novel substitutions including S63P, D143E, A183V, A207T, G208K, G208A, G208E, I212S, E214K, and M191I were identified in the *gyrA* of either/both fluoroquinolone-resistant or/and -susceptible strains in this study. Among them, mutations M191I, G208E, and V199A were predominantly found in fluoroquinolone-resistant isolates. Moreover, *gyrB* mutations may rarely occur and have little impact on primary fluoroquinolone resistance [5,8,42,45,65,68]. In this study, only two amino acid changes, D481E and R484K, were identified in *gyrB*, in which R484K was exclusively present in resistant strains.

Among macrolides, clarithromycin is recognized as a major antibiotic for *H. pylori* eradication therapy because of its impact on treatment outcomes [20,69]. The rate of clarithromycin resistance is topically much lower than that of metronidazole. However, the rate of primary clarithromycin resistance is undoubtedly on the rise and varies between different geographical regions [7,8,47,57,70]. Unfortunately, the level of clarithromycin resistance in this study increased in comparison with a previous study from 26% to 33.8%, which is of great concern [35].

It has been claimed that the three most frequently reported mutations, including A2143G, A2142G, and A2142C, are responsible for more than 90% of the cases of primary resistance to clarithromycin [71,72]. However, in a recent study by De Francesco et al., this concordance was reduced to only 54.8%, with the A2142C mutation not being detected at all [20]. Moreover, some other mutations have been found to be associated with clarithromycin resistance, although their precise role is not yet clear [73]. In this study, only four mutations including A2143G and C2195T were found in our isolates. In contrast, Khashei et al. reported that A2142G was the most (90%) frequent point mutation among the isolates from southwest of Iran a PCR-RFLP method [74]. In addition, we failed to identify additional mutations such as T2183C and A2223G, which are frequently reported to be the cause of clarithromycin resistance in Eastern countries, rather than in Western countries [75]. Additionally, no point mutation was identified in the sequence of the 23S rRNA gene in four clarithromycin-resistant strains. For those isolates, we can speculate that other resistance mechanisms, such as the presence of an efflux pump, may be implicated in the development of resistance to clarithromycin [76].

It is estimated that the overall resistance rates to amoxicillin and tetracycline are 23.61% and 7.38% in Asian countries, respectively [52]. Similarly, we observed a high rate of resistance to these drugs among the studied isolates (30.9% to amoxicillin and 4.4% to tetracycline), which is a matter of great concern in *H. pylori* eradication in Iran. However, the level of resistance to these antibiotics was reported to be very low or even absent in most Western countries versus African countries [73,77]. Regarding rifampicin, we also observed a rising rate of resistance from 14.4% to 32.4% in comparison with a previous report [35]. Recently, Regnath et al. reported a considerable increase in resistance to rifampicin from 3.9% to 18.8% between 2002 and 2015 among pediatric patients from southwest Germany [78].

Unfortunately, the emergence of MDR *H. pylori* strains has become a serious challenge all over the world. In a previous study, the resistance rate to at least two antimicrobial agents was reported in 43% of the *H. pylori* isolates from Iran [35]. Surprisingly, our finding showed that 75% of the isolates were resistant to at least two antibiotics. The high prevalence of MDR phenotype may be attributed to the exhaustive use of antibiotics across the country. Information about the prevalence of quadruple-drug resistance is limited, and a few reports from India (2.5%), Bulgaria (0.7%), Vietnam (1.9%), and Indonesia (2.6%) are available thus far [5,47,79,80]. However, 16.2% of the isolates in this study showed quadruple-drug resistance, which was lower than the previous study (37.9%) [35]. Moreover, resistance to tetracycline was only observed in the isolates with quadruple-drug resistance. This finding could be explained by the presence of multidrug efflux pumps in these strains [73].

There have been several reports on the relationship between *H. pylori* virulence markers and antibiotic resistance. Accordingly, patients infected with *cagA*-positive strains that also carry more virulent *vacA* alleles have significantly high cure rates and eradication success compared with less virulent strains [26,81,82,83]. It has been hypothesized that the colonization of gastric mucosa by more virulent *H. pylori* genotypes may induce a higher degree of inflammation and increase blood flow, which in turn can favor better diffusion of the antibiotics [27,83]. Alternatively, another possible explanation may be related to the fact that *cagA*-positive strains proliferate faster than *cagA*-negative strains, and would thus be more susceptible to antibiotics [25,81]. Furthermore, Taneike et al. observed that *cagA*-negative strains may tend to acquire spontaneous drug resistance under selective pressure of antimicrobials [25]. In contrast, we found a significant association between the *cagA*-positive genotype and resistance to MNZ (*p* = 0.0002). However, it still remains somewhat controversial because recent reports indicated that these virulent genotypes variously distributed between susceptible and resistant strains [5,28,84,85,86]. CagA protein with a greater number of EPIYA-C repeats are considered to be pathophysiologically more virulent and carcinogenic [87]. Thus, according to the above-mentioned hypothesis, we expected the presence of more virulent types of CagA EPIYA motifs in susceptible isolates than resistant ones. However, as the number of EPIYA types having two or more EPIYA-C repeats was very low, we could not come to such a conclusion.

*H. pylori* strains that carry an intact and functional *cag*PAI are more virulent and frequently associated with severe clinical outcomes than those carrying partial or no *cag*PAI [31,39]. As far as we know, this is the first study that relates the *cag*PAI integrity with antibiotic resistance. Our results showed that *H. pylori* isolates harboring intact or partial *cag*PAI were variably distributed between susceptible and resistant isolates. However, most of the resistant isolates to the antibiotic tested and all isolates resistant to TCN carried an intact *cag*PAI locus. Similar to other studies performed in Italy (37.2%) [88], North Wales (53%) [89], and Germany (37.4%) [82], *vacA* s1m2 (51.5%) genotype was the most prevalent *vacA* mosaicisms in our strains. Although *H. pylori* strains with *vacA* s1m2 were detected more frequently in resistant isolates, no significant associations were found (*p* > 0.05). Moreover, in our study, the majority of resistant isolates had *oipA* “on” status and were positive for *babA2*, *sabA,* and *dupA* genotypes. However, we found no statistically significant association between these virulence factors and antibiotics resistance (*p* > 0.05), except for the *oipA* “on” status and resistance to AMX (*p* = 0.02), and *sabA*- and *dupA*-positivity with resistance to MNZ (*p* = 0.0001). These results are contradictory and did not strongly support the idea that susceptibility to antibiotics is higher in infections caused by more virulent genotypes. Nevertheless, it is likely that infected patients with resistant and hypervirulent strains are at increased risk of progression to more severe clinical outcomes owing to failure in *H. pylori* eradication.

## 5. Conclusions

In conclusion, this study demonstrated that the prevalence of *H. pylori* antibiotic resistance is worrisome in our country with rising trends over the time. The findings from this study also highlight the relevance of different types of mutations in genes responsible for antibiotic resistance in *H. pylori* strains. We also provide evidence for the importance of simultaneous screening of the virulence and resistance genotypes in *H. pylori* strains for guiding clinicians to choose an appropriate combination of drugs. Taken together, because of the alarming increase in the rate of *H. pylori* antibiotic resistance in our local population, it is reasonable to constantly monitor the antimicrobial susceptibility patterns, and develop effective treatment and preventive strategies at the national level.

## Figures and Tables

**Table 1:** Oligonucleotide primer sequences used for amplification of genes involved in *H. pylori* antibiotic resistance.

Target Gene	Primer Designation	Oligonucleotide Sequence (5′–3′)	Annealing Temperature (°C)	PCR Product (bp)	Reference
*cagA*	93089 93261	AATACACCAACGCCTCCAAG TTGTTGCCGCTTTTGCTCTC	57	400	[37]
*cagL*	CagL-B4 CagL-B5	GCAGAATTCATAACAAGCGGCTTAAAG ATTAGAATTCATAGCCTATCGTCTCAG	60	695	[37]
*vacA* s1/s2	VA1-F VA1-R	ATGGAAATACAACAAACACAC CTGCTTGAATGCGCCAAAC	57	259/286	[37]
*vacA* m1/m2	VAG-F VAG-R	CAATCTGTCCAATCAAGCGAG GCGTCAAAATAATTCCAAGG	57	570/645	[37]
*babA2*	Bab7-F Bab7-R	CCAAACGAAACAAAAAGCGT GCTTGTGTAAAAGCCGTCGT	52	271	[37]
*sabA*	F1-HP726-jhp663 R1-HP725-jhp662	TTTTTGTCAGCTACGCGTTC ACCGAAGTGATAACGGCTTG	55	581	[37]
*oipA*	OipA-F OipA-R	CAAGCGCTTAACAGATAGGC AAGGCGTTTTCTGCTGAAGC	56	450	[40]
*dupA*	DupA-F DupA-R	ATTCACGCCTAAGACCTCA CTGAGAAGCCTTATTATCTTGTTGG	52	487	[38]
*frxA*	frx1 frx2	TGGATATGGCAGCCGTTTA GGTTATCAAAAAGCTAACAGCG	52	729	[41]
*rdxA*	rdx1 rdx2	ATGGTAATTGTTTCGTTAGGG CTCCTTGAACTTTAATTTAG	48	758	[41]
*gyrA*	gyrAPF gyrAPR	AGCTTATTCCATGAGCGTGA TCAGGCCCTTTGACAAATTC	52	582	[42]
*gyrB*	gyrBPF gyrBPR	CCCTAACGAAGCCAAAATCA GGGCGCAAATAACGATAGAA	51	465	[42]
23S rRNA	Hp23-1 Hp23-2	CCACAGCGATGTGGTCTCAG CTCCATAAGAGCCAAAGCCC	54	425	[43]

**Table 2:** Distribution of the antibiotic resistance patterns, minimal inhibition concentration (MIC) range, MIC_50_ values, and MIC_90_ values for each antibiotic among *H. pylori* isolates used in this study.

Antibiotic Agents	MIC Range	MIC_50_	MIC_90_	No. (%) of MIC (mg/L)	
				Susceptible	Resistant
MNZ	0.25–128	32	64	12 (17.6)	56 (82.4)
CLR ^a^	0.06–16	0.125	8	40 (58.8)	23 (33.8)
AMX	0.03–0.5	0.06	0.5	47 (69.1)	21 (30.9)
CIP	0.06–32	0.5	16	45 (66.2)	23 (33.8)
LEV	0.03–32	0.25	8	49 (72.1)	19 (27.9)
RIF	0.03–32	0.5	8	46 (67.6)	22 (32.4)
TCN	0.06–4	0.125	0.5	65 (95.6)	3 (4.4)

Abbreviations: MNZ, metronidazole; CLR, clarithromycin; AMX, amoxicillin; CIP, ciprofloxacin; LEV, levofloxacin; RIF, rifampicin; TCN, tetracycline; MIC, minimal inhibition concentrations. ^a^ Five (7.3%) *H. pylori* isolates had intermediate susceptibility against clarithromycin based on CLSI breakpoints (MIC values equal to 0.5 mg/L).

**Table 3:** Distribution of MIC values for each antibiotic among *H. pylori* isolates used in this study.

MIC (mg/L)	No. (%)						
	MNZ	CLR	AMX	CIP	LEV	RIF	TCN
0.03	NA	NA	24 (35.3)	NA	5 (7.3)	5 (7.3)	NA
0.06	NA	25 (36.8)	15 (22)	3 (4.4)	4 (5.9)	9 (13.2)	17 (25)
0.125	NA	11 (16.2)	8 (11.8)	17 (25)	10 (14.7)	15 (22)	32 (47)
0.25	4 (5.9)	4 (5.9)	12 (17.6)	13 (19.1)	15 (22)	4 (5.9)	7 (10.3)
0.5	ND	5 (7.3)	9 (13.2)	5 (7.3)	6 (8.8)	8 (11.8)	5 (7.3)
1	3 (4.4)	5 (7.3)	ND	7 (10.3)	9 (13.2)	5 (7.3)	4 (5.9)
2	ND	6 (8.8)	ND	8 (11.8)	7 (10.3)	9 (13.2)	2 (2.9)
4	ND	3 (4.4)	ND	3 (4.4)	4 (5.9)	2 (2.9)	1 (1.5)
8	5 (7.3)	2 (2.9)	NA	2 (2.9)	3 (4.4)	5 (7.3)	ND
16	16 (23.5)	7 (10.3)	NA	9 (13.2)	4 (18.2)	4 (5.9)	ND
32	14 (20.6)	ND	NA	1 (1.5)	1 (1.5)	2 (2.9)	NA
64	19 (27.9)	ND	NA	NA	NA	NA	NA
128	7 (10.3)	NA	NA	NA	NA	NA	NA
256	ND	NA	NA	NA	NA	NA	NA

Abbreviations: MNZ, metronidazole; CLR, clarithromycin; AMX, amoxicillin; CIP, ciprofloxacin; LEV, levofloxacin; RIF, rifampicin; TCN, tetracycline. NA, not applicable; ND, not detected.

**Table 4:** Distribution of the multidrug resistance profiles in relation to clinical outcomes among *H. pylori* isolates used in this study.

Resistance Profiles	Clinical Diagnosis			Total No. (%)
	CG (*n* = 42)	PUD (*n* = 18)	IM (*n* = 7)	
**Single drugs**				
CLR	2	1	0	3 (4.5)
AMX	1	0	0	1 (1.5)
RIF	2	0	0	2 (3)
CIP	0	1	0	1 (1.5)
MNZ	6	2	1	9 (13.4)
**Double drugs**				
MNZ + AMX	3	2	0	5 (7.5)
MNZ + RIF	4	1	1	6 (8.9)
CIP + RIF	3	1	0	4 (6)
MNZ + CIP	2	1	0	3 (4.5)
MNZ + LEV	1	1	0	2 (3)
**Triple drugs**				
MNZ + CLR + LEV	1	2	1	4 (6)
MNZ + AMX + RIF	3	1	0	4 (6)
MNZ + CLR + CIP	2	1	1	4 (6)
MNZ + CLR + AMX	1	0	1	2 (3)
MNZ + AMX + CIP/LEV	2	1	0	3 (4.5)
MNZ + RIF + CIP/LEV	3	0	0	3 (4.5)
**Quadruple drugs**				
MNZ + CLR + AMX + LEV	2	1	0	3 (4.5)
MNZ + CLR + AMX + CIP/LEV	0	0	1	1 (1.5)
MNZ + CLR + TCN + LEV	1	1	0	2 (3)
MNZ + TCN + RIF + LEV	0	0	1	1 (1.5)
MNZ + CLR + AMX + CIP	1	1	0	2 (3)
MNZ + CLR + CIP + RIF	2	0	0	2 (3)

Abbreviations: MNZ, metronidazole; CLR, clarithromycin; AMX, amoxicillin; CIP, ciprofloxacin; LEV, levofloxacin; RIF, rifampicin; TCN, tetracycline; CG, chronic gastritis; PUD, peptic ulcer disease; IM, intestinal metaplasia.

**Table 5:** Number of nucleotide insertion and deletion in *frxA* and *rdxA* genes involved in metronidazole resistance among *H. pylori* isolates used in this study.

Strains	Metronidazole Resistance Phenotype	MIC (mg/L)	No. of Nucleotide ins/del	Mutations	No. of Nucleotide ins/del	Mutation	SDR/MDR	Clinical Diagnosis
			*frxA*		*rdxA*			
OC80	Susceptible	0.25	None ^a^	In-frame	None ^a^	In-frame	N ^c^	CG
OC112	Resistant	32	ND ^b^	ND ^b^	None ^a^	In-frame	MDR	CG
HC114	Resistant	16	Ins (2)/Del (4)	Frameshift	None ^a^	In-frame	SDR	PUD
HC138	Resistant	32	Del (2)	Frameshift	None ^a^	In-frame	MDR	PUD
HC168	Resistant	32	Del (1)	Frameshift	ND ^b^	ND ^b^	MDR	PUD
OC179	Resistant	64	ND ^b^	ND ^b^	ND ^b^	ND ^b^	SDR	CG
OC180	Resistant	32	Del (2)	Frameshift	Del (4)	Frameshift	MDR	IM
OC217	Resistant	32	Del (1)	Frameshift	Ins (1)	Frameshift	MDR	CG
OC218	Susceptible	0.25	ND ^b^	ND ^b^	ND ^b^	ND ^b^	SDR	CG
OC235	Resistant	32	Del (2)	Frameshift	Ins (9)	Frameshift	MDR	PUD
OC245	Resistant	8	ND ^b^	ND ^b^	ND ^b^	ND ^b^	MDR	IM
OC250	Susceptible	1	None ^a^	In-frame	None ^a^	In-frame	SDR	PUD
OC485	Resistant	128	Del (1)	Frameshift	None ^a^	In-frame	MDR	CG
OC494	Susceptible	0.25	None ^a^	In-frame	None ^a^	In-frame	MDR	CG
OC557	Resistant	64	Del (3)	Frameshift	None ^a^	In-frame	MDR	PUD
OC562	Susceptible	8	None ^a^	In-frame	None ^a^	In-frame	SDR	CG
OC571	Resistant	64	None ^a^	In-frame	None ^a^	In-frame	MDR	CG
OC576	Resistant	64	Del (1)	Frameshift	Q50Stop	PTC	SDR	CG
OC688	Resistant	16	Del (1)	Frameshift	Q50Stop	PTC	MDR	IM
OC797	Resistant	16	Del (1)	Frameshift	None ^a^	In-frame	MDR	IM
OC803	Resistant	16	None ^a^	In-frame	None ^a^	In-frame	MDR	CG
OC810	Resistant	64	None ^a^	In-frame	Del (1)	Frameshift	MDR	CG
OC824	Resistant	128	None ^a^	In-frame	Q50Stop	PTC	MDR	PUD
OC840	Susceptible	1	None ^a^	In-frame	None ^a^	In-frame	SDR	PUD
OC852	Resistant	16	ND ^b^	ND ^b^	Ins (4)	Frameshift	MDR	IM
OC897	Resistant	16	None ^a^	In-frame	Ins (6)/Del (2)	Frameshift	SDR	PUD
OC912	Resistant	16	Del (1)	Frameshift	Del (1)	Frameshift	MDR	PUD
OC913	Resistant	128	Ins (3)/Del (1)	Frameshift	None ^a^	In-frame	MDR	PUD
OC937	Resistant	128	ND ^b^	ND ^b^	Del (1)	Frameshift	SDR	CG
OC939	Resistant	64	Q5Stop	PTC	None ^a^	In-frame	MDR	PUD
OC975	Resistant	64	None ^a^	In-frame	ND ^b^	ND ^b^	MDR	IM
OC985	Resistant	64	None ^a^	In-frame	Del (1)	Frameshift	MDR	CG
OC1031	Resistant	128	Ins (2)	Frameshift	Del (1)	Frameshift	MDR	CG

Abbreviations: Ins, nucleotide insertion; Del, nucleotide deletion; PTC, premature termination codon; SDR, single-drug resistance; MDR, multidrug resistance; CG, chronic gastritis; PUD, peptic ulcer disease; IM, intestinal metaplasia. ^a^ None, no specific variation detected as compared with genes or amino acids from metronidazole-sensitive *H. pylori* isolates; ^b^ ND, not determined (the obtained sequence was not appropriate for mutational analysis); ^c^ N, not resistant to any of the antibiotics tested.

**Table 6:** Mutations in *gyrA* and *gyrB* genes involved in fluoroquinolone resistance among *H. pylori* isolates used in this study.

Strains	Resistance Phenotype CIP/LEV	MIC (mg/L)		Mutations		SDR/MDR	Clinical Diagnosis
		CIP	LEV	*gyrA*	*gyrB*		
OC80	Susceptible/Susceptible	0.5	0.06	M191I, V199A, G208E	D481E	N ^c^	CG
OC112	Susceptible/Susceptible	0.25	0.25	M191I, G208E	None ^a^	MDR	CG
HC114	Susceptible/Susceptible	0.12	0.12	M191I, G208E	None ^a^	SDR	PUD
HC138	Resistant/Susceptible	16	0.5	V150A, M191I, G208E	None ^a^	MDR	PUD
HC168	Susceptible/Susceptible	0.25	0.06	D143E, G208K, I212S, E214K	ND ^b^	MDR	PUD
OC179	Susceptible/Susceptible	0.5	0.06	M191I, V199A, G208A	ND ^b^	SDR	CG
OC180	Susceptible/Susceptible	0.12	0.06	V150A, M191I, V199A, G208E	None ^a^	MDR	IM
OC217	Susceptible/Susceptible	0.12	0.06	M191I, G208E	ND ^b^	MDR	CG
OC218	Susceptible/Susceptible	0.12	0.06	ND ^b^	ND ^b^	SDR	CG
OC235	Susceptible/Susceptible	0.25	0.06	D86N, M191I, G208E	None ^a^	MDR	PUD
OC245	Resistant/Resistant	16	16	M191I, V199A, G208A	None ^a^	MDR	IM
OC250 ^d^	Resistant/Susceptible	16	0.5	D86G, M191I	None ^a^	SDR	PUD
OC485 ^d^	Resistant/Susceptible	2	0.12	D86N, A183V, M191I	None ^a^	MDR	CG
OC494 ^d^	Resistant/Susceptible	32	0.5	None ^a^	None ^a^	MDR	CG
OC557	Susceptible/Resistant	0.12	16	V199A, G208E	None ^a^	MDR	PUD
OC562	Susceptible/Susceptible	1	0.12	D86G, M191I, A207T, G208E	ND ^b^	SDR	CG
OC571	Resistant/Resistant	16	16	D86G, M191I, V199A, G208E	None ^a^	MDR	CG
OC576	Susceptible/Susceptible	0.06	0.5	V199A, G208E	D481E	SDR	CG
OC688	Susceptible/Susceptible	1	1	M191I, V199A, G208E	None ^a^	MDR	IM
OC797	Resistant/Susceptible	2	0.03	M191I, V199A, G208E	D481E, R484K	MDR	IM
OC803	Resistant/Susceptible	2	0.03	S63P, M191I, V199A, G208E	None ^a^	MDR	CG
OC810	Susceptible/Resistant	1	32	M191I, G208E	None ^a^	MDR	CG
OC824	Resistant/Resistant	16	16	M191I, G208E	D481E, R484K	MDR	PUD
OC840	Susceptible/Susceptible	1	0.06	G208E	None ^a^	SDR	PUD
OC852	Susceptible/Resistant	0.5	2	M191I, G208E	D481E, R484K	MDR	IM
OC897	Susceptible/Susceptible	0.25	0.03	A97V, G208E	None ^a^	SDR	PUD
OC912	Resistant/Susceptible	2	0.12	G208E	D481E, R484K	MDR	PUD
OC913	Susceptible/Resistant	0.25	16	M191I, G208E	D481E, R484K	MDR	PUD
OC937	Susceptible/Susceptible	0.12	0.5	G208E	None ^a^	SDR	CG
OC939	Resistant/Susceptible	4	0.12	M191I, G208E	None ^a^	MDR	PUD
OC975	Susceptible/Resistant	0.25	16	D86N, R140K, M191I, G208E	None ^a^	MDR	IM
OC985	Susceptible/Susceptible	0.12	0.06	ND ^b^	None ^a^	MDR	CG
OC1031	Resistant/Resistant	16	16	D86N, M191I, G208E	ND ^b^	MDR	CG

Abbreviations: CIP, ciprofloxacin; LEV, levofloxacin; SDR, single-drug resistance; MDR, multidrug resistance; CG, chronic gastritis; PUD, peptic ulcer disease; IM, intestinal metaplasia. ^a^ None, no specific variation detected as compared with genes or amino acids from fluoroquinolone-sensitive *H. pylori* isolates; ^b^ ND, not determined (the obtained sequence was not appropriate for mutational analysis); ^c^ N, not resistant to any of the antibiotics tested; ^d^ the *gyrA* quinolone-resistant determining regions of the strains OC250, OC485, and OC494 were partially translated because of the low quality of the obtained sequences.

**Table 7:** Mutations in the 23S rRNA gene involved in clarithromycin resistance among *H. pylori* isolates used in this study.

Strains	Clarithromycin Resistance Phenotype	MIC (mg/L)	Mutations	SDR/MDR	Clinical Diagnosis
OC80	Susceptible	0.062	None ^a^	N ^c^	CG
OC112	Susceptible	0.125	ND ^b^	MDR	CG
HC114	Susceptible	0.062	None ^a^	SDR	PUD
HC138	Resistant	16	None ^a^	MDR	PUD
HC168	Susceptible	0.062	None ^a^	MDR	PUD
OC179	Susceptible	0.062	ND ^b^	SDR	CG
OC180	Resistant	16	A2143G	MDR	IM
OC217	Susceptible	0.062	ND ^b^	MDR	CG
OC218	Susceptible	0.25	ND ^b^	SDR	CG
OC235	Intermediate	0.5	None ^a^	MDR	PUD
OC245	Resistant	16	ND ^b^	MDR	IM
OC250	Susceptible	0.25	None ^a^	SDR	PUD
OC485	Resistant	2	None ^a^	MDR	CG
OC494	Susceptible	0.062	None ^a^	MDR	CG
OC557	Resistant	2	C2195T	MDR	PUD
OC562	Intermediate	0.5	ND ^b^	SDR	CG
OC571	Susceptible	0.25	None ^a^	MDR	CG
OC576	Susceptible	0.125	ND ^b^	SDR	CG
OC688	Susceptible	0.125	None ^a^	MDR	IM
OC797	Resistant	16	None ^a^	MDR	IM
OC803	Resistant	1	ND ^b^	MDR	CG
OC810	Resistant	2	C2195T	MDR	CG
OC824	Susceptible	0.062	None ^a^	MDR	PUD
OC840	Resistant	1	A2143G	SDR	PUD
OC852	Resistant	2	ND ^b^	MDR	IM
OC897	Susceptible	0.062	None ^a^	SDR	PUD
OC912	Intermediate	0.5	None ^a^	MDR	PUD
OC913	Susceptible	0.125	None ^a^	MDR	PUD
OC937	Susceptible	0.125	None ^a^	SDR	CG
OC939	Resistant	4	None ^a^	MDR	PUD
OC975	Susceptible	0.125	None ^a^	MDR	IM
OC985	Susceptible	0.125	None ^a^	MDR	CG
OC1031	Susceptible	0.062	None ^a^	MDR	CG

Abbreviations: SDR, single-drug resistance; MDR, multidrug resistance; CG, chronic gastritis; PUD, peptic ulcer disease; IM, intestinal metaplasia. ^a^ None, no specific variation detected as compared with genes from clarithromycin-sensitive *H. pylori* isolates; ^b^ ND, not determined (the obtained sequence was not appropriate for mutational analysis); ^c^ N, not resistant to any of the antibiotics tested.

**Table 8:** Frequency and distribution of virulence genotypes in relation to antibiotic resistance patterns among *H. pylori* isolates used in this study.

Virulence Genotypes	Resistance No. (%)															Total No. (%)
	MNZ		CLR			AMX		CIP		LEV		RIF		TCN		
	S (*n* = 12)	R (*n* = 56)	S (*n* = 40)	I (*n* = 5)	R (*n* = 23)	S (*n* = 47)	R (*n* = 21)	S (*n* = 45)	R (*n* = 23)	S (*n* = 49)	R (*n* = 19)	S (*n* = 46)	R (*n* = 22)	S (*n* = 65)	R (*n* = 3)	
*cagL^+^*	11 (16.2)	55 (80.9)	38 (55.9)	5 (7.5)	23 (33.8)	46 (67.6)	20 (29.4)	43 (63.2)	23 (33.8)	47 (69.1)	19 (27.9)	44 (64.7)	22 (32.3)	63 (92.6)	3 (4.4)	66/68 (97)
*cagL¯*	1 (1.5)	1 (1.5)	2 (2.9)	0	0	1 (1.5)	1 (1.5)	2 (2.9)	0	2 (2.9)	0	2 (2.9)	0	2 (2.9)	0	2/68 (2.9)
*cagA^+^*	5 (7.5)	52 (76.5)	33 (48.5)	4 (5.9)	20 (29.4)	39 (57.3)	18 (26.5)	36 (52.9)	21 (30.9)	40 (58.8)	17 (25)	38 (55.9)	19 (27.9)	54 (79.4)	3 (4.4)	57/68 (83.8)
*cagA¯*	7 (10.4)	4 (5.9)	7 (10.4)	1 (1.5)	3 (4.4)	8 (11.8)	3 (4.4)	9 (13.2)	2 (2.9)	9 (13.2)	2 (2.9)	8 (11.8)	3 (4.4)	11 (16.2)	0	11/68 (16.2)
***cagA* EPIYA motifs**																
ABC	3 (5.3)	36 (63.1)	24 (42.1)	2 (3.5)	13 (22.8)	29 (50.9)	10 (17.5)	28 (49.1)	11 (19.3)	27 (47.4)	12 (21)	27 (47.4)	12 (21)	37 (64.9)	2 (3.5)	39/57 (68.4)
ABCC	1 (1.7)	3 (5.3)	2 (3.5)	0	2 (3.5)	1 (1.7)	3 (5.3)	1 (1.7)	3 (5.3)	3 (5.3)	1 (1.7)	2 (3.5)	2 (3.5)	4 (7))	0	4/57 (7)
ABCCC	0	2 (3.5)	1 (1.7)	0	1 (1.7)	0	2 (3.5)	0	2 (3.5)	1 (1.7)	1 (1.7)	2 (3.5)	0	1 (1.7)	1 (1.7)	2/57 (3.5)
Mixed type ^a^	1 (1.7)	11 (19.3)	6 (10.5)	2 (3.5)	4 (7)	9 (15.8)	3 (5.3)	7 (12.3)	5 (8.8)	9 (15.8)	3 (5.3)	7 (12.3)	5 (8.8)	12 (21)	0	12/57 (21)
***cag*PAI integrity**																
Intact *cag*PAI	5 (7.5)	39 (58.2)	24 (35.2)	3 (4.5)	17 (25.4)	32 (47.8)	12 (17.9)	26 (38.8)	18 (26.9)	31 (46.3)	13 (22.8)	26 (38.8)	18 (26.9)	41 (61.2)	3 (4.5)	44/67 (65.7)
Partial *cag*PAI	7 (10.4)	16 (23.9)	15 (22.4)	2 (3)	6 (8.9)	14 (20.9)	9 (13.4)	18 (26.9)	5 (7.5)	17 (25.4)	6 (8.9)	19 (28.3)	4 (5.9)	23 (34.3)	0	23/67 (34.3)
Totally deleted *cag*PAI	0	1 (1.5)	1 (1.5)	0	0	1 (1.5)	0	1 (1.5)	0	1 (1.5)	0	1 (1.5)	0	1 (1.5)	0	1/68 (1.5)
***vacA* alleles**																
*vacA* s1m1	7 (10.4)	23 (33.8)	14 (20.6)	2 (2.9)	10 (14.7)	18 (26.5)	8 (11.8)	17 (25)	9 (13.2)	19 (27.9)	7 (10.4)	18 (26.5)	8 (11.8)	25 (36.8)	1 (1.5)	26/68 (38.2)
*vacA* s1m2	4 (5.9)	29 (42.6)	22 (32.3)	2 (2.9)	11 (16.2)	26 (38.2)	9 (13.2)	24 (35.3)	11 (16.2)	26 (38.2)	9 (13.2)	23 (33.8)	12 (17.6)	33 (48.5)	2 (2.9)	35/68 (51.5)
*vacA* s2m2	1 (1.5)	4 (5.9)	4 (5.9)	1 (1.5)	2 (2.9)	3 (4.4)	4 (5.9)	4 (5.9)	3 (4.4)	4 (5.9)	3 (4.4)	5 (7.5)	2 (2.9)	7 (10.4)	0	7/68 (10.3)
**Adhesions**																
Oip “on”	10 (14.7)	49 (72)	37 (54.4)	3 (4.4)	19 (27.9)	44 (64.7)	15 (22)	38 (55.9)	21 (30.9)	42 (61.8)	17 (25)	40 (58.8)	19 (27.9)	57 (83.8)	2 (2.9)	59/68 (86.8)
Oip “off”	2 (2.9)	7 (10.4)	3 (4.4)	2 (2.9)	4 (5.9)	3 (4.4)	6 (8.8)	7 (10.4)	2 (2.9)	7 (10.4)	2 (2.9)	6 (8.8)	3 (4.4)	8 (11.8)	1 (1.5)	9/68 (13.2)
*babA2^+^*	10 (14.7)	55 (80.9)	37 (54.4)	5 (7.5)	23 (33.8)	45 (66.2)	20 (29.4)	43 (63.2)	22 (32.3)	46 (67.6)	19 (27.9)	44 (64.7)	21 (30.9)	62 (91.2)	3 (4.4)	65/68 (95.6)
*babA2¯*	2 (2.9)	1 (1.5)	3 (4.4)	0	0	2 (2.9)	1 (1.5)	2 (2.9)	1 (1.5)	3 (4.4)	0	2 (2.9)	1 (1.5)	3 (4.4)	0	3/68 (4.4)
*sabA^+^*	3 (4.4)	52 (76.5)	32 (47)	4 (5.9)	19 (27.9)	38 (55.9)	17 (25)	36 (52.9)	19 (27.9)	41 (60.3)	14 (20.6)	37 (54.4)	18 (26.5)	52 (76.5)	3 (4.4)	55/68 (80.9)
*sabA¯*	9 (13.2)	4 (5.9)	8 (11.8)	1 (1.5)	4 (5.9)	9 (13.2)	4 (5.9)	9 (13.2)	4 (5.9)	8 (11.8)	5 (7.5)	9 (13.2)	4 (5.9)	13 (19.1)	0	13/68 (19.1)
*dupA^+^*	5 (7.5)	53 (77.9)	34 (50)	4 (5.9)	20 (29.4)	40 (58.8)	18 (26.5)	38 (55.9)	20 (29.4)	42 (61.8)	16 (23.5)	39 (57.3)	19 (27.9)	56 (82.3)	2 (2.9)	58/68 (85.3)
*dupA¯*	7 (10.4)	3 (4.4)	6 (8.8)	1 (1.5)	3 (4.4)	7 (10.4)	3 (4.4)	7 (10.4)	3 (4.4)	7 (10.4)	3 (4.4)	7 (10.4)	3 (4.4)	9 (13.2)	1 (1.5)	10/68 (14.7)

Abbreviations: MNZ, metronidazole; CLR, clarithromycin; AMX, amoxicillin; CIP, ciprofloxacin; LEV, levofloxacin; RIF, rifampicin; TCN, tetracycline; S, susceptible; I, intermediate; R, resistant, ^a^ Denotes the presence of multiple cagA EPIYA motifs, indicating mixed infections.

## Supplementary Materials

The following are available online at https://www.mdpi.com/2077-0383/8/11/2004/s1, Figure S1: Amino acid sequence alignment of FrxA in metronidazole-susceptible and -resistant *H. pylori* isolates (*n* = 27) as compared with *H. pylori* reference strain 26695. The stop codon and premature truncation in peptide translation is indicated with (#); Figure S2: Amino acid sequence alignment of RdxA in metronidazole-susceptible and -resistant *H. pylori* isolates (*n* = 28) as compared with *H. pylori* reference strain 26695. The stop codon and premature truncation in peptide translation is indicated with (#); Figure S3: Amino acid sequence alignment of GyrA in fluoroquinolone-susceptible and -resistant *H. pylori* isolates (*n* = 31) as compared with *H. pylori* reference strain 26695; Figure S4: Amino acid sequence alignment of GyrB in fluoroquinolone-susceptible and -resistant *H. pylori* isolates (*n* = 27) as compared with *H. pylori* reference strain 26695; Figure S5: Nucleotide sequence alignment of 23S rRNA in clarithromycin-susceptible and -resistant *H. pylori* isolates (*n* = 24) as compared with *H. pylori* reference strain 26695.
