# Supplementary material for: High Prevalence of Antibiotic Resistance in Iranian Helicobacter pylori Isolates: Importance of Functional and Mutational Analysis of Resistance Genes and Virulence Genotyping
Source: J Clin Med. 2019 Nov 17;8(11):2004. doi: 10.3390/jcm8112004 (PMC6912791; doi:10.3390/jcm8112004)
Supplement: Supplementary file 1 [file jcm-08-02004-s001.pdf]

Supplementary Figure 1:

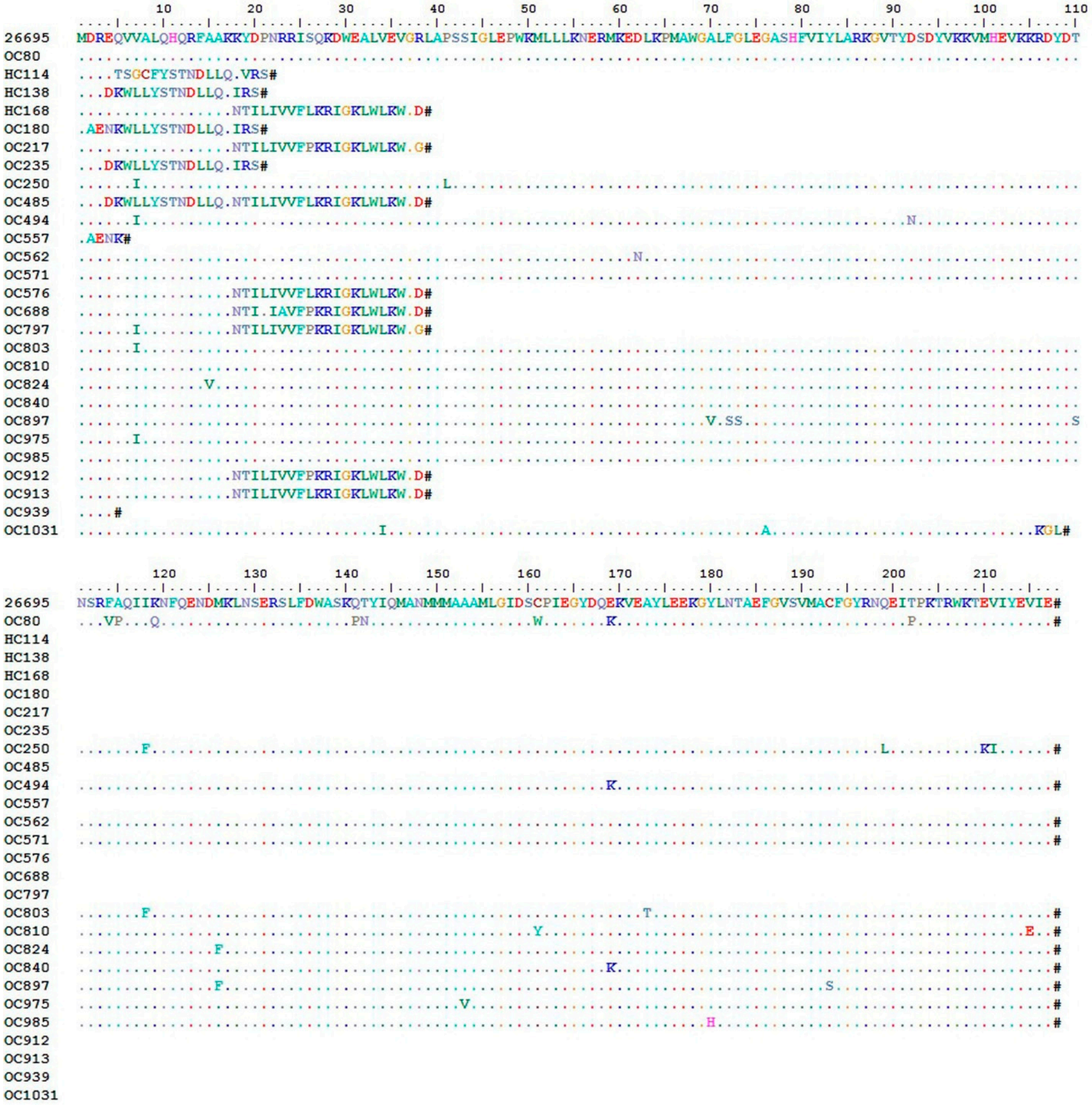

Figure S1. Amino acid sequence alignment of FrxA in metronidazole-susceptible and -resistant *H. pylori* isolates (n = 27) as compared with *H. pylori* reference strain 26695. The stop codon and premature truncation in peptide translation is indicated with (#).

Supplementary Figure 2:

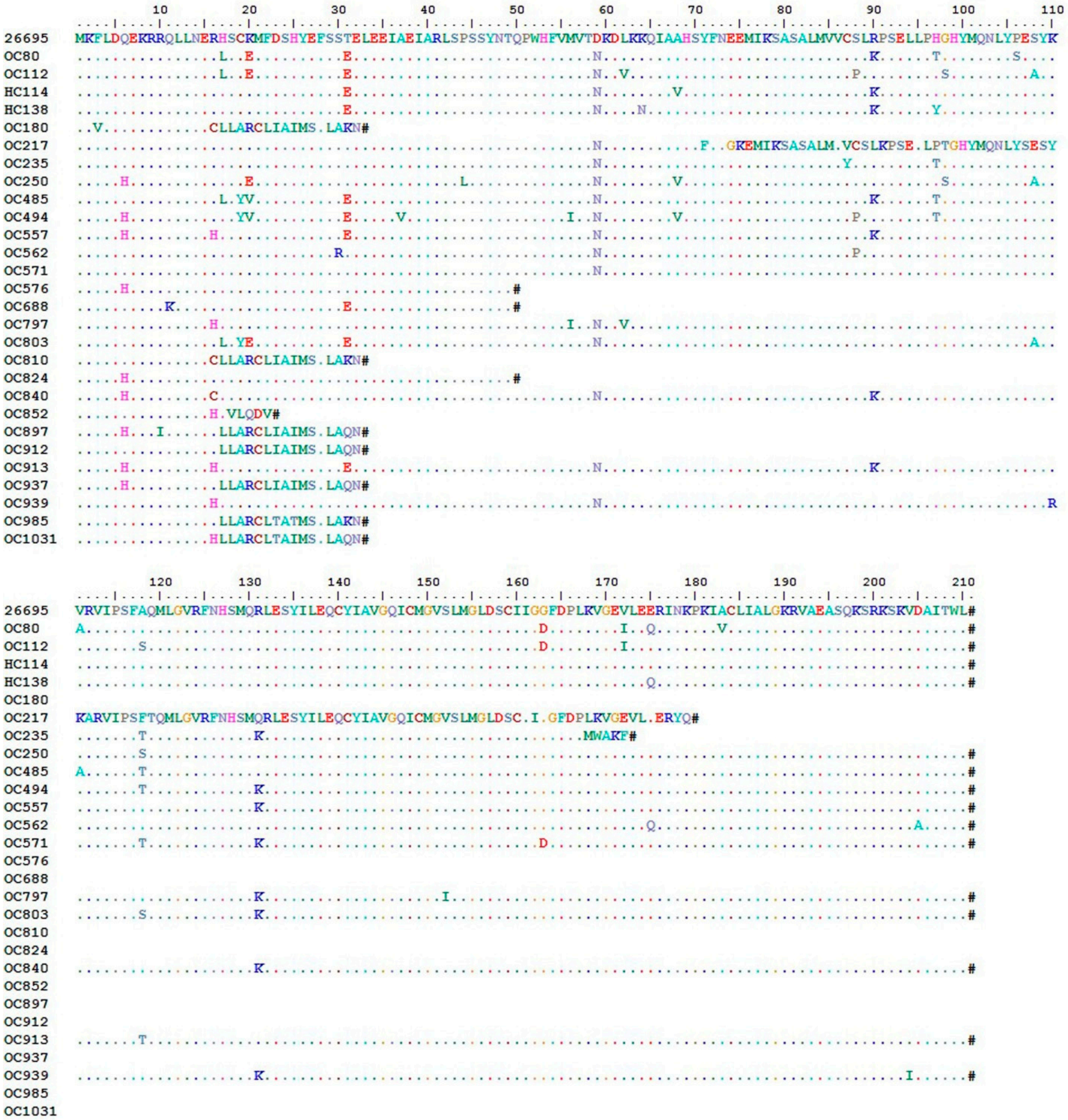

Figure S2. Amino acid sequence alignment of RdxA in metronidazole-susceptible and -resistant *H. pylori* isolates (n = 28) as compared with *H. pylori* reference strain 26695. The stop codon and premature truncation in peptide translation is indicated with (#).

Supplementary Figure 3:

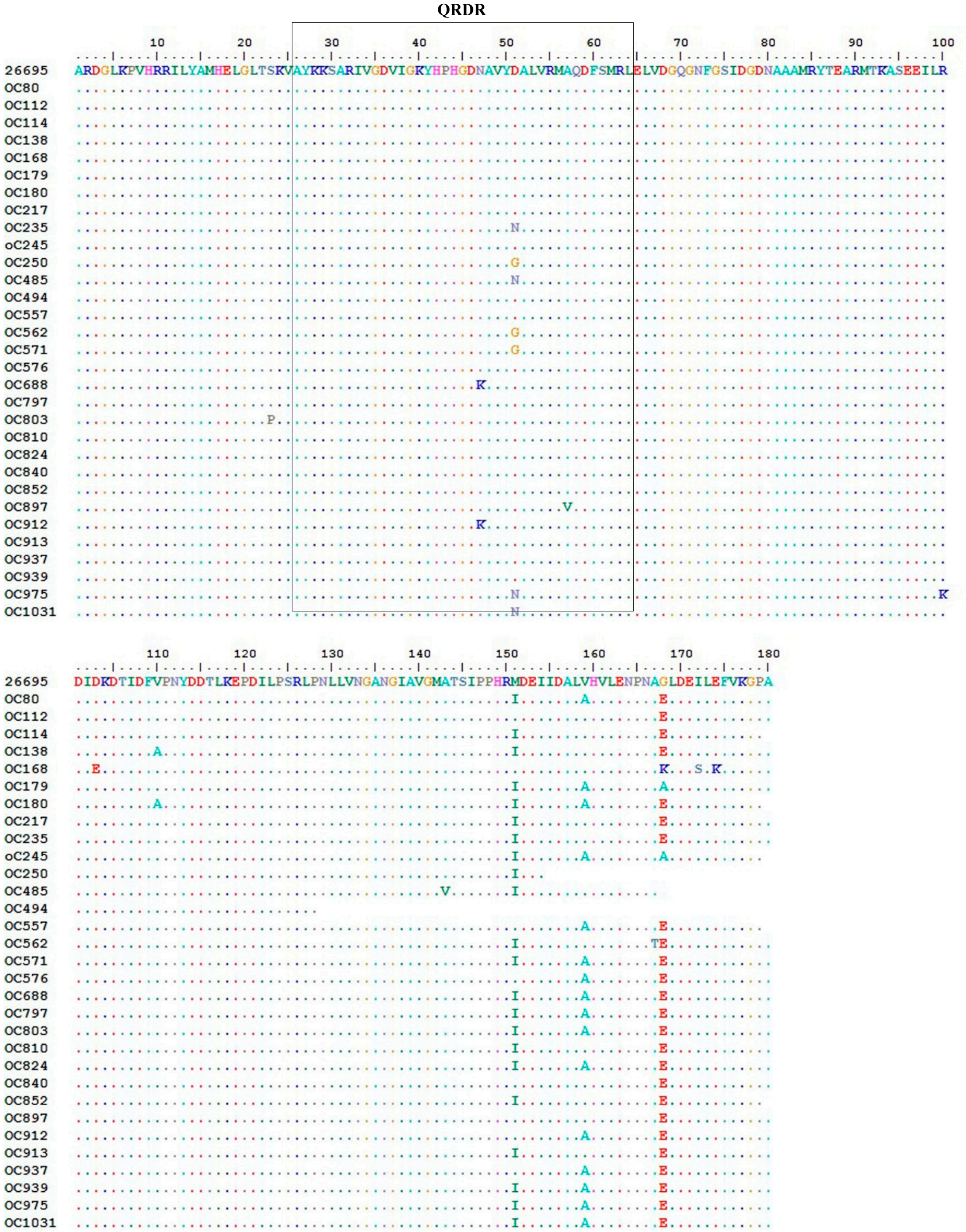

Figure S3. Amino acid sequence alignment of GyrA in fluoroquinolone-susceptible and -resistant *H. pylori* isolates (n = 31) as compared with *H. pylori* reference strain 26695.

Supplementary Figure 4:

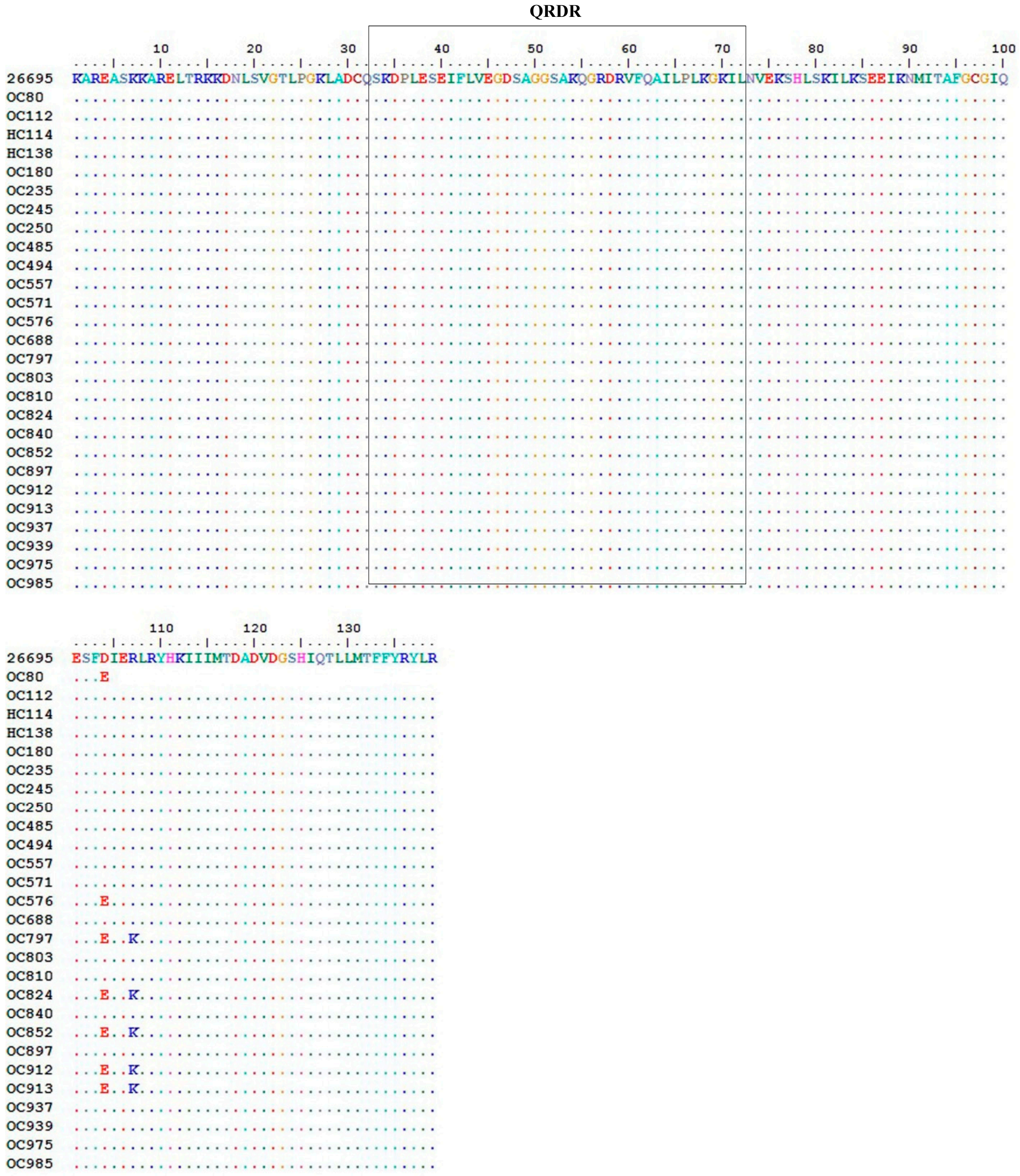

Figure S4. Amino acid sequence alignment of GyrB in fluoroquinolone-susceptible and -resistant *H. pylori* isolates (n = 27) as compared with *H. pylori* reference strain 26695.

Supplementary Figure 5:

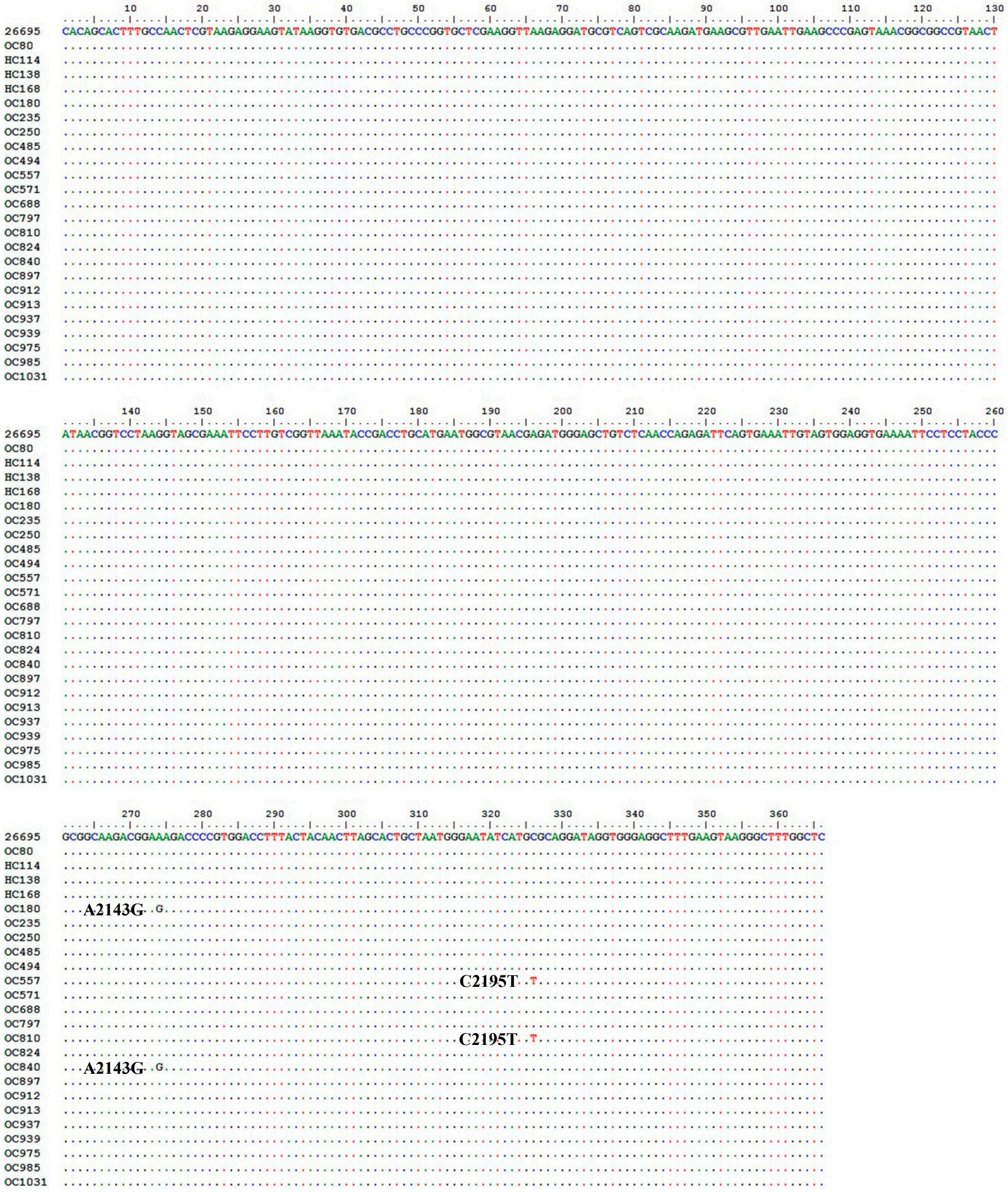

Figure S5. Nucleotide sequence alignment of 23S rRNA in clarithromycin-susceptible and -resistant *H. pylori* isolates (n = 24) as compared with *H. pylori* reference strain 26695.
